# Comparing the ability of molecular diagnosis and CAP-inhibition in identifying the really causative venom in patients with positive tests to Vespula and Polistes species

**DOI:** 10.1186/s12948-016-0040-5

**Published:** 2016-02-08

**Authors:** Eleonora Savi, Silvia Peveri, Elena Makri, Valerio Pravettoni, Cristoforo Incorvaia

**Affiliations:** Allergy Unit, G. Da Saliceto Hospital, AUSL Piacenza, Piacenza, Italy; Allergy/Pulmonary Rehabilitation, ICP Hospital, Via Bignami 1, 20100 Milan, Italy; Department of Internal Medicine, Clinical Allergy and Immunology, IRCCS Foundation Ca’ Granda Ospedale Maggiore Policlinico, Milan, Italy

**Keywords:** Hymenoptera venom allergy, Vespids, Diagnosis, CAP inhibition, Molecular allergy

## Abstract

**Background:**

Cross-reactivity among Hymenoptera venoms is an important issue when prescribing venom immunotherapy (VIT). Using all venoms eliciting a positive response results in treatment excess and unjustified cost increase. The first in vitro method that helped to identify the really causative venom was RAST-inhibition, but in latest years also molecular allergy (MA) diagnostics, that detects specific sIgE to single venom allergens, was introduced. We compared the two methods in patients with double sensitization to *Vespula* spp. and *Polistes* spp.

**Methods:**

Fifty-four patients with anaphylactic reactions to Hymenoptera stings and positive results to skin tests and sIgE measurement with whole venom from *Vespula* spp. and *Polistes dominula* were included in the study. Sera from all patients were analyzed by CAP-inhibition (Thermo Fisher Scientific, Uppsala, Sweden) and MA diagnostics with recombinant Ves v 1, Ves v 5 and Pol d 5.

**Results:**

By the data obtained from MA technique, VIT would have been prescribed to 7 patients for *Polistes*, to 6 for *Vespula*, and to 41 for both venoms. With the data from CAP inhibition, it would have been a prescription to 15 patients for *Polistes*, to 28 for *Vespula*, and to 11 for both venoms. A good concordance between the results of MA and CAP-inhibition was found only when the value in kU/l of Ves v 5 were about twice those of Pol d 5, and vice versa.

**Conclusions:**

These findings suggest that in the choice of the venom to be used for VIT CAP-inhibition remains a pivotal tool, because the significance of in vitro inhibition is definite and provides a diagnostic importance higher than MA in patients with positive tests to both *Vespula* and *Polistes* spp.

## Background

Stings by Hymenoptera, including honeybees (*Apis mellifera*), yellow jackets (*Vespula species*), paper wasps (*Polistes species*), and hornets (*Dolicho vespula*, *Vespa crabro*) cause systemic allergic reactions in 1–5 % of the population in Europe and North America [1]. The mechanism of these reactions is an IgE-mediated sensitization to proteins of the venoms injected with the stings, particularly enzymes like phospholipase A and hyaluronidase, and for vespids antigen 5 [2]. A common issue in diagnosis of Hymenoptera venom allergy (HVA) is the occurrence of multiple positive results to the different venoms, mainly due to cross-reactivity between phospholipase A1 for vespids, and hyaluronidase, that may concern all venoms; another important cross-reactivity source is common cross-reactive carbohydrate determinants (CCD) [3]. This has led to perform very often venom immunotherapy (VIT) with all venoms eliciting a positive response to tests. However, in the 1990s Hamilton et al. demonstrated by the technique of RAST-inhibition that one third of 305 patients with HVA with positive *Vespul*a- and *Polistes*-reactive IgE in the skin and/or serum were identified as candidates for exclusion of *Polistes* from immunotherapy because their IgE anti-*Polistes* was more than 95 % cross-inhibitable with *Vespula* venom [4]. After the demonstration that CAP system had better performances than RAST [5], CAP-inhibition was used instead to assess cross-reactivity. Concerning Hymenoptera venom, CAP-inhibition studies allowed to detect the importance of CCD as a cause for the double positivity to *A. mellifera* and *Vespula* venom [6], and the extent of cross-reactivity between *Vespa crabro* and *Vespula* venom [7]. Two studies focused the role of CAP-inhibition in suggesting the correct choice of the venom to be used for VIT in patients with apparent poly-sensitization [8, 9].

In the latest decade, the technique of molecular allergy (MA), that makes possible to measure specific IgE (sIgE) to single venom allergens was introduced as a further in vitro method to identify with precision the causative venom [10]. In fact, in patients with positive tests to *A. mellifera* and *Vespula* venom the detection of sIgE to Api m 1, Ves v 1 and Ves v 5 allowed recognition of double sensitization from cross-reactions [11]. Indeed, double positivity to tests with *Vespula* and *Polistes species* is very frequent. In 2012, a MA-based study on patients with such kind of sensitization found that *Polistes* and *Vespula* were the culprit insect in 49 and 20 %, respectively [12], but this was in contrast with the general knowledge on the importance of these species in HVA [13].

The aim of this study was to compare the capacity of MA technique and CAP-inhibition to identify the culprit vespid in patients positive to both *Vespula* and *Polistes* whole venoms.

## Methods

Fifty-four outpatients with anaphylactic reactions to Hymenoptera stings of at least Mueller grade II [14] in the last 6 months and double positive response to tests with *Vespula* and *Polistes* spp. were included in the study. All patients did not recognize the culprit insect and thus were unable to provide data to identify the really causative species. Skin tests were performed with *Vespul*a spp. and *Polistesdominula* venom (ALK-Abellò Horsholm, Denmark) for *Vespula species* and *P. dominula,* with an initial prick test at 100 mcg/ml, followed, if negative, by intradermal testing at 0.1 and 1 mcg/ml [15]. Venom-specific serum IgE measurement was done by CAP system (Thermo Fisher Scientific, Uppsala, Sweden) to whole venom of *Vespula* spp. and *P. dominula*, as well to recombinant Ves v 1, Ves v 5 and Pol d 5.

All patients sample sera were analyzed by CAP-inhibition to identify the actual sensitization by the laboratory method previously described by Caruso et al. [9]. Briefly, two 100 mcL aliquots of patient’s serum were incubated separately for 12 h at 4 °C with 200 ml of *P. dominula* or *Vespula* venoms at increasing dilutions (0; 25 mcg; 50 mcg; 100 mcg/ml). Subsequently, sIgE against each of the venoms were determined in the samples prepared as above. The extent of homologous (blockage of venom-sIgE by the same venom) and heterologous (blockage of the venom-sIgE by the other venom) inhibition was computed with the following formula:  % inhibition = 100 − [IgE inhibited sample (kU/l) × 100/IgE anti-venom (kU/l) at zero concentration of venom]. According to Straumann et al. [8] a percentage of homologous inhibition >70 % was required to perform heterologous inhibition. The same percentage was considered suggestive of cross-reactivity among *Vespula* and *Polistes* venom. The venom preparations tested were the same used for skin tests. For statistical analysis was used the Cohen (k) concordance index, that establishes the grade of concordance based on the following k values: <0, no concordance, 0–0.4 poor concordance, 0.4–0.6, fair concordance, 0.6–0.8, good concordance, and 0.8–1, excellent concordance.

All patients gave their consent to the use of the data obtained from the diagnostic procedures for scientific research.

## Results

The patients had similar reactivity by skin testing to *Vespula* and *Polistes* spp. venom. An homologous inhibition higher than 70 % was detected in all sera. Table 1 shows the data obtained from measurement of sIgE to whole venom by CAP and to single allergens by MA technique. By such data, based on MA results VIT would have been prescribed to 7 patients with *Polistes*, to 6 patients with *Vespula*, and to 41 patients with both venoms. Based on CAP inhibition data VIT would have prescribed to 15 patients with *Polistes*, to 28 patients with *Vespula*, and to 11 patients with both venoms. The concordance between the results of MA with Ves v 1, Ves v 5, Pol d 5 and the results of CAP-inhibition was very poor, showing a value of k = 0.01. A good concordance was found only when the value in kU/l of Ves v 5 were about twice those of Pol d 5, and vice versa.

**Table 1:** Results obtained with MA technique and CAP-inhibition and choice of VIT for each method. The same choices for MA and CAP-inhibition are highlighted in italics

No	Age/sex	Whole venom extracts		Recombinant allergens (kUA/L)			Venom used for VIT		CAP inhibition (% of inhibition)				Venom used for VIT	
		*Vespula* spp.	*Polistes dominula*	Ves v 5	Ves v 1	Pol d 5	*Vespula*	*Polistes*	*Vespula* spp. slgE *Vespula* spp. venom	*P. dominula* slgE *Vespula* spp. venom	*Vespula* spp. slgE by *P.* *dominula* venom	*P. dominula* slgE by *P. dominula* venom	*Vespula*	*Polistes*
1	45/m	5.54	4.65	1.15	3	0.57	*X*		88	72	80	87	*X*	
2	56/f	5.14	9.3	47.2	3	81.4	*X*	*X*	80	50	35	78	*X*	*X*
3	68/f	1.73	1.94	9.66	0	14.6	X	X	100	35	80	92		X
4	57/m	0.63	1.41	1.7	1	2.59	*X*	*X*	100	50	50	80	*X*	*X*
5	74/m	>100	>100	5.21	0.09	4.28	X	X	88	80	65	90	X	
6	76/m	55.3	80.3	17.3	41.3	17.6	X	X	75	35	70	95		X
7	35/m	2.51	1.17	17.5	0.04	21.4	*X*	*X*	80	50	50	77	*X*	*X*
8	61/m	1.28	2.62	2.93	0	5.57	X	X	95	50	95	92		X
9	48/m	9.49	1.99	1.88	7.31	1.17	X	X	95	75	55	82	X	
10	71/m	6.04	1.73	12.1	1.68	8.73	X	X	100	73	40	78	X	
11	52/m	10.4	7.36	10.5	10.7	11.1	X	X	75	73	82	93		X
12	62/m	0.53	1.08	2.47	0	4.44	X	X	95	60	97	99		X
13	23/m	13.3	9.76	14.9	6	8.34	*X*	*X*	90	40	40	90	*X*	*X*
14	45/m	1.89	1.93	1.48	1.8	1.93	*X*	*X*	90	60	65	86	*X*	*X*
15	44/f	3.65	1.34	20.1	0.06	18.1	*X*	*X*	93	35	27	90	*X*	*X*
16	64/f	2.59	1.73	32.3	0.01	38.7	X	X	99	86	78	93	X	
17	66/m	2.2	10.1	3.8	0.42	7.28	*X*	*X*	93	35	35	92	*X*	*X*
18	42/m	29.2	46.3	5.31	24.9	22.2		*X*	95	60	87	94		*X*
19	58/m	3.2	3.82	5.72	1.6	4.26	*X*	*X*	80	58	50	85	*X*	*X*
20	34/m	6.23	5.79	4.36	4.31	3.76	X	X	75	69	55	90	X	
21	55/f	4.54	9.71	5.86	0.74	10.2	*X*	*X*	74	33	50	80	*X*	*X*
22	55/m	13.8	7.48	12.8	4.33	5.19	*X*		90	75	33	82	*X*	
23	43/m	7.71	6.45	2.78	22.2	0.94	*X*		88	80	65	88	*X*	
24	51/f	6.37	7.16	6.5	0.02	7.4	X	X	94	80	73	93	X	
25	67/m	1.82	1.79	0.4	0.78	0.39	X	X	94	70	60	92	X	
26	72/m	5.52	12.7	4.49	0.92	9.93		*X*	86	50	87	75		*X*
27	75/m	4.88	5.39	3.97	0.05	4.77	X	X	75	63	79	94		X
28	55/m	75.6	1.3	76.8	1.11	66.1	X	X	98	96	70	75	X	
29	57/f	5.35	2.33	4.2	0	1.3	*X*		96	94	30	78	*X*	
30	66/m	3.14	02:34	2.89	0.04	0.04	*X*		99	92	54	80	*X*	
31	47/m	40	32	99	0.17	98	X	X	83	93	36	78	X	
32	65/f	8.16	5.15	7.1	2.3	4.6	X	X	98	99	56	75	X	
33	59/m	3.51	3	1.73	0.7	2	X	X	84	78	52	79	X	
34	60/m	1.43	1.2	46	0.1	4.23	X	X	97	70	67	76	X	
35	21/m	1.6	2.3	1.9	0.46	2	*X*	*X*	95	66	61	93	*X*	*X*
36	55/m	6.04	5.93	5.18	0.82	5.17	X	X	97	70	57	75	X	
37	46/f	1.35	3.11	5.89	0.02	10.8	X	X	92	83	59	83	X	
38	32/f	1.05	1.83	0.88	0.8	2.25		*X*	76	45	82	76		*X*
39	50/f	4.01	2.33	94.8	9.22	81.4	X	X	99	96	64	87	X	
40	47/m	1.2	1.8	3.14	0	5.48	X	X	85	40	80	75		X
41	56/m	2.14	2.98	27.6	0.39	45.2	X	X	92	86	75	76	X	
42	41/m	>100	>100	99	0.16	98.5	X	X	99	95	60	92	X	
43	51/f	1.74	1.7	0.6	0.08	0.59	X	X	90	81	52	76	X	
44	74/m	19.2	17.5	16.8	0.19	19.2	X	X	95	85	47	85	X	
45	45/m	1.06	2.27	1.3	0.09	5.8		*X*	94	44	91	91		*X*
46	67/m	22.8	83.6	21	18	63		*X*	95	42	96	98		*X*
47	25/m	3.9	5.59	1.5	2.3	5.6		*X*	75	30	65	70		*X*
48	22/m	5.02	12	5.49	0.92	19.93		*X*	86	50	87	75		*X*
49	68/m	4.15	3.38	5.6	1.3	1.6	*X*		90	65	45	78	*X*	
50	59/f	3.83	5.02	3.53	0.1	4.98	X	X	96	52	83	90		X
51	61/m	17.3	15.8	15.8	0.3	14.3	X	X	98	76	59	86	X	
52	63/m	6.15	8.9	7.03	8.14	9.45	*X*	*X*	96	54	61	90	*X*	*X*
53	16/m	5.46	3.76	6.08	3.92	6.18	X	X	81	83	29	82	X	
54	52/f	1.33	1.34	0.56	0.05	0.75	X	X	94	86	37	75	X	

## Discussion

A number of studies demonstrated that VIT prevents any kind of reaction to insect stings in most patients and is completely effective in preventing fatal reactions [16]. To expect the same outcome in the daily practice, the allergist must choice the appropriate venom for VIT, but this is often complicated by the issue of cross-reactivity, which concerns all venoms but is particularly common for vespids [13]. The first in vitro technique that helped to identify the really causative venom was RAST-inhibition, followed by CAP-inhibition, whose reagents allow more accurate measurements [5–8]. In recent years the technique of MA diagnostics was introduced and detects the IgE antibodies to single molecules contained in allergen sources [17]. Assessing sIgE to recombinant and natural venom components from each vespid species in 45 patients with allergic reactions to stings and positive ImmunoCAP and/or intradermal tests to vespid venoms, Monsalve et al. found that 9 of these patients had clearly higher IgE values to nVes v 1 or nVes v 5 or both, thus indicating that *Vespula* was most probably the sensitizing species for these patients, while the probable sensitization could be clearly assigned to *P. dominula* in 22/45 cases, because of the higher values of Pol d 1 and/or Pol d 5. In 14/45, the quantitative response did not allow to identify the possible sensitizing species [12]. For such cases, the authors suggested that, “unless complex inhibition studies or sting challenges are performed”, double sensitization should be considered to prescribe a correct immunotherapy. More recently, Hemmer stated that “The identification of the primary venom in patients testing positive for *Vespula* and *Polistes* (paper wasps) is particularly important in Mediterranean areas. MA technique with the marker allergens Ves v 5 and Pol d 5 may directly identify the causative venom in the majority of patients” [18].

Actually, our findings show that MA would have indicated 7 treatments with *Polistes* and 6 with *Vespula*, while information obtained by CAP-inhibition would have indicated 15 treatments with *Polistes* and 28 with *Vespula*, thus reducing the number of double treatments from 41 to 11, and confirming the observation from Hamilton et al. on the capacity of this in vitro method to avoid unnecessary VIT treatments [4]. This suggests that in the choice of the venom to be used in immunotherapy CAP-inhibition remains a pivotal tool, because the significance of in vitro inhibition is definite and provides a diagnostic importance higher than MA in patients with positive tests to both *Vespula* and *Polistes* spp. who failed to recognize the culprit vespid. The availability of additional molecules, such as Pol d 1, will probably improve the ability of MA in vespid allergy, but this warrants to be demonstrated by further studies. Both MA and CAP-inhibition studies thus far available were not tested against a gold standard for diagnosis of HVA. Indeed, differently from other fields of allergy, for example with the double-blind placebo-controlled food challenge for the diagnosis of food allergy [19], no gold standard is definitely accepted for HVA. In particular, the sting challenge test is not recommended for vespid allergy due to the variable amounts of venom injected with the sting [20]. The actual demonstration of the tolerance to venom induced by VIT is based on the outcome of field stings, but follow-up studies investigating the proof of the results of MA or CAP-inhibition by field stings are not available.

Indeed, in times of spending review also the cost-effectiveness of VIT is questioned [21]. Nobody can argue its life-saving role when the culprit venom is used, but avoiding additional treatments with unnecessary venoms, as obtained by CAP-inhibition, that in our study spared 30 additional treatments in 54 patients, may allow to significantly reduce the costs.
